# Systems-Level G Protein-Coupled Receptor Therapy Across a Neurodegenerative Continuum by the GLP-1 Receptor System

**DOI:** 10.3389/fendo.2014.00142

**Published:** 2014-09-01

**Authors:** Jonathan Janssens, Harmonie Etienne, Sherif Idriss, Abdelkrim Azmi, Bronwen Martin, Stuart Maudsley

**Affiliations:** ^1^Translational Neurobiology Group, VIB Department of Molecular Genetics, University of Antwerp, Antwerp, Belgium; ^2^Metabolism Unit, National Institute on Aging, National Institutes of Health, Bethesda, MD, USA

**Keywords:** transcriptomics and proteomics, pharmacotherapeutics, pathophysiology, heptahelical G protein-coupled receptor

## Abstract

With our increasing appreciation of the true complexity of diseases and pathophysiologies, it is clear that this knowledge needs to inform the future development of pharmacotherapeutics. For many disorders, the disease mechanism itself is a complex process spanning multiple signaling networks, tissues, and organ systems. Identifying the precise nature and locations of the pathophysiology is crucial for the creation of systemically effective drugs. Diseases once considered constrained to a limited range of organ systems, e.g., central neurodegenerative disorders such as Alzheimer’s disease (AD), Parkinson’s disease (PD), and Huntington’ disease (HD), the role of multiple central and peripheral organ systems in the etiology of such diseases is now widely accepted. With this knowledge, it is increasingly clear that these seemingly distinct neurodegenerative disorders (AD, PD, and HD) possess multiple pathophysiological similarities thereby demonstrating an inter-related continuum of disease-related molecular alterations. With this systems-level appreciation of neurodegenerative diseases, it is now imperative to consider that pharmacotherapeutics should be developed specifically to address the systemic imbalances that create the disorders. Identification of potential systems-level signaling axes may facilitate the generation of therapeutic agents with synergistic remedial activity across multiple tissues, organ systems, and even diseases. Here, we discuss the potentially therapeutic systems-level interaction of the glucagon-like peptide 1 (GLP-1) ligand–receptor axis with multiple aspects of the AD, PD, and HD neurodegenerative continuum.

## Introduction

Heptahelical G protein-coupled receptors (GPCRs) represent perhaps the most important single-protein class of pharmacotherapeutic targets. GPCR systems have adapted to perceive almost all forms of environmental and endogenous signaling entities, e.g., photons, odorants, lipids, carbohydrates, peptides, and nucleic acids. Currently, nearly 50% of existing licensed therapeutic compounds functionally interact with GPCRs (1, 2). Not only do GPCRs constitute one of the primary therapeutic targets but also their importance in systemic biology cannot be underestimated, as GPCRs constitute approximately 5% of the genome in early species such as the nematode worm and still approximately 1.5% of the genome in *Homo sapiens* (3–5). Therefore, it is likely that the molecular diversity is closely associated with changes in organismal complexity and compartmentalization. Rudimentary organisms such as nematode worms, with little tissue separation or definition, often require multiple ligands and receptors to specify particular signaling modalities. However, with the increased tissue complexity and physical separation present in highly complex multisystem organisms such as *Homo sapiens*, this ligand–receptor multiplicity has been refined to achieve the same degree of functional specificity. Therefore, instead of producing a single ligand–receptor system for each distinct function, ligand–receptor systems have been reproduced in distinct and often distant sites but are functionally differentiated from each other via cell-type specific scaffolding and transduction system interactions (6–13). Such a paradigm therefore creates a whole-organism mechanism to coordinate the activities of ligand–receptor systems at multiple tissue sites. While simple organisms essentially can constitute one physical interaction environment, more complex organisms require the subtly regulated behavior of multiple tissue/organ systems to be present for the homeostasis of such complex events as energy metabolism or reproduction. It is now clear from recent research that more complex communication systems, which overarch traditional organ axis [e.g., the hypothalamic–pituitary–gonadal (HPG) axis], potentially exist and that these “super-axes” are instead coordinated and generated around repeated and transposed ligand–receptor systems (14–20). The generation of these ligand–receptor super-axes therefore demonstrates an alternative method compared to more clinically based anatomical axes, in which to study and appreciate both physiological and pathophysiological processes. While normal homeostatic processes can be coordinated through these systems, it is likely that disease processes as well could be specifically present at this level of molecular complexity. While these added dimensions of physiological appreciation may seem daunting, they do, however, present the capacity to identify ligand–receptor super-axes that can be targeted for therapeutic situations in which a wide-scope of therapeutic activity is desired (6). It is in this context that our review is focused, i.e., is there potential ligand–receptor super-axis therapeutics that can ameliorate the multiple central and peripheral pathological processes that are present in neurodegenerative diseases such as Alzheimer’s disease (AD), Parkinson’s disease (PD), and Huntington’s disease (HD). In addition to the identification of potential super-axis therapeutics, we shall also discuss the potential strategies for engineered specificity of functional activity and signaling bias.

## Systems-Level Expression of Neurodegenerative Disorders

In recent years, considerable evidence has accumulated that demonstrates that many of the classical central neurodegenerative disorders also coincide with significant and widespread peripheral pathophysiologies. In this respect, we must start to consider that diseases such as AD, PD, and HD may all share some common and coordinated pathological axes that are present in both central and peripheral sites. The presence of such a controlling system provides the possibility for a mechanism to systemically regulate and eventually remediate these diseases in a whole-body-wide approach. In the following sections, we will outline the multiple commonalities across these diseases and then allude to potential therapeutic systems that may facilitate such a multi-level therapy.

## Alzheimer’s Disease

Alzheimer’s disease (AD) is the most common form of dementia that currently affects over 30 million people worldwide. With increasing life-span and lack of interventions to slow or stop the progression of AD, epidemiological studies indicate that this number is expected to reach 115.4 million in 2050 (21–25). Two different forms of AD have been described: the inherited form called “early-onset familial AD” (FAD) is caused by rare genetic variations in genes encoding amyloid precursor protein (APP), presenilin 1 (PSEN1), and 2 (PSEN2) involved in β-amyloid (Aβ) processing and which affect the intracellular trafficking of Aβ or APP (26, 27). Since <1% of AD cases are caused by genetic variations (27), the vast majority of AD cases are affected by the “sporadic late-onset” form of AD (SAD) (26, 27), which remains poorly understood. No genetic cause is known for SAD, but numerous risk factors exist such as aging, which is considered the most common risk factor for developing AD, head trauma, traumatic brain injury, metabolic dysfunctions, and many others. Furthermore, the ε4 allele of apolipoprotein E (APOE) represents another very important risk factor in both FAD and SAD (28, 29). The APOE protein is crucial for phospholipid and cholesterol transport and for neuronal damage repair. AD-related neurodegeneration causes progressive memory loss and a decline in cognitive abilities. These symptoms are associated with two neuropathological lesions: senile plaques that correspond to an extracellular deposition of Aβ peptides (27, 28), and neurofibrillary tangles (NFTs) due to the intracellular accumulation of the hyperphosphorylated microtubule protein Tau (30–32). AD-related behavioral abnormalities result from neuronal dysfunctions and death in different brain regions involved in cognition and mood such as the hippocampus, the amygdala, and the temporal, parietal, and frontal lobes of the cerebral cortex (25, 33, 34). AD pathology also includes coexisting metabolic and hormonal dysfunctions, inflammatory processes, excitotoxic damage, altered energy metabolism, and oxidative stress, not only in the brain but also throughout the body. These metabolic dysfunctions observed throughout the body are likely to contribute as well to neurological symptoms observed in AD (26, 27). Therefore, it is now clearly established that we need to take in consideration many other somatic and metabolic dysfunctions present in AD for a better understanding of the disease and to provide new targets for novel preventive or therapeutic efficacious treatments.

### Metabolic dysfunction in AD

Metabolic syndrome (MetS) is a disorder of energy utilization and storage and is characterized by abdominal obesity, elevated blood pressure, increased plasma glucose levels, high serum triglycerides, and low high-density cholesterol (HDL) levels (27, 28). Consequently, MetS has been considered as a risk factor for the development of cardiovascular diseases, type 2 diabetes mellitus (T2DM), and a number of dementias such as AD in patients over age 75 years (27). This is also reflected by the cellular and biochemical alterations observed in MetS since some of them have also been observed in AD patients (27, 28). In addition to MetS, insulin resistance in peripheral tissues corresponds to one of the main syndromes in T2DM and constitutes a high risk factor of developing AD (35). Insulin resistance results in the incapacity of insulin, generated in pancreatic β-cells, to mediate effective glucose uptake. Epidemiological studies have reported that people with diabetes mellitus have a 1.5- to 2.5-fold greater risk of developing cognitive impairment and dementia (36, 37). Moreover, hyperglycemia and insulin resistance are also likely to deteriorate the neuropathology of AD, especially in APOE4 carriers. Mechanistically, the increased risk of dementia with T2DM or obesity could be due to hyperglycemia, peripheral insulin resistance, oxidative stress, accumulation of advanced glycation end products, increased production of pro-inflammatory cytokines, and cerebral microvascular disease (38).

### Inflammatory processes in AD

Alzheimer’s disease is also closely associated with low-grade chronic inflammation (39) as highlighted by a number of increased systemic inflammation markers including C-reactive protein (CRP), fibrinogen, interleukin-6 (IL-6), and tumor necrosis factor α (TNF-α) (39, 40). It is well established that chronic inflammation in AD associated with MetS may arise from a deregulation of the endocrine homeostasis of adipose tissue, which is infiltrated by immune cells and produces pro-inflammatory molecules such as adipokines, cytokines, and chemokines (40). Pro-inflammatory molecules inhibit mitochondrial respiration in the brain, decrease the activities of the electron transport chain and mitochondrial membrane potential, increase mitochondrial membrane permeability, enhance reactive oxygen species (ROS) production, interfere with ATP production and cause mitochondrial shutdown, ultimately leading to neuronal degeneration (41). Increased levels of inflammatory molecules such as interleukin-1 (IL-1), IL-6, TNF-α, CRP, granulocyte macrophage colony-stimulating factor (GM-CSF), eotaxin, and macrophages inflammatory protein 1-α (MIP-1 α) have been reported in brain tissue from patients with AD (40, 42). In addition, 18 plasma biomarkers related to inflammation had been identified in patients affected with AD (23). Chronic inflammation is characterized by mononuclear cell infiltration of the affected tissue, angiogenesis, tissue clearance, fibrosis, and tissue remodeling that can interfere with the optimal tissue functions (43) and lead to some pathological disorders. Moreover, chronic inflammation may play a role in accelerated cognitive impairment by a direct effect on the brain or by influencing the development of vascular disease (36).

### AD and gastrointestinal function

In addition to alterations of the central neuronal activity, it is now clear that a similar degradation of the enteric nervous system (ENS) in AD is apparent (44, 45). Gastrointestinal (GI) motility deficits are frequently occurring co-morbidities in dementia and represent a long-term debilitating problem. The structural integrity of gut muscle as well as the innervating ENS is both subject to amyloid-related cellular toxicity. Disruption of gut motility and the ability to absorb nutrients will significantly reduce metabolic support for stressed tissues across the whole body as nutrient absorption will be curtailed. With respect to GI energy uptake, alterations of multiple pancreatic factors are also involved in AD pathology, including amylin, leptin, and ghrelin. Amylin and leptin are both hormones that activate overlapping intracellular signaling pathways such as STAT3, Akt, and AMPK, which have a role in glucose and lipid metabolism and have an additive effect in signaling pathways. Amylin is co-localized and co-secreted with insulin from pancreatic β-cells in response to nutrient stimuli. As insulin secretion, the secretion of amylin is also impaired in T2DM. As for the deposition of Aβ in AD, amylin is also an amyloidogenic protein (islet amyloid peptide), which gets deposited in T2DM indicating that both AD and T2DM share many common disease features (46). In addition to disruption of the gut activity itself, there are also additional effects of AD pathology on the gut–brain axis communication. The orexigenic peptide ghrelin is primarily produced by the stomach, controls the central hunger sensation, and forms one of the major components of the gut–brain axis (26, 47). Ghrelin levels are often altered in AD patients suggesting that the gut–brain axis may contribute to defective cognition (48, 49). Ghrelin exerts an opposite effect to the anorexigenic leptin on food intake and energy homeostasis (26). Interestingly, it has been demonstrated that intracerebrovascular injection of ghrelin improved cognitive ability in streptozotocin-induced diabetic-rats by increasing the expression of cAMP response element-binding protein (CREB) and brain-derived neurotrophic factor (BDNF), and by attenuating the neuronal apoptosis in the hippocampus (27, 50). These findings suggested that ghrelin plays a pivotal role in metabolic control but also in regulating cognitive function and memory capacity.

### AD and adipose tissue

While insulin increases the production of leptin by adipose tissue, leptin exerts a negative feedback on both insulin secretion and insulin gene expression in pancreatic islet β-cells. These effects are mediated by both the autonomic nervous system and by direct actions via leptin receptors on pancreatic β-cells (51, 52). AD-related insulin resistance syndromes could be associated with the dysfunction of the adipoinsular axis leading to obesity and hyperinsulinemia (26). Leptin is directly neuroprotective in dopaminergic cells, potentially via APP- and tau-related mechanisms (26). Adiponectin is a protein hormone specifically released from adipose tissue and regulates the sensitivity of insulin, modulates fatty acid catabolism, glucose homeostasis, and anti-inflammatory systems. Adiponectin plays also a role in memory and cognitive impairment and contributes to a dysregulated glucose metabolism and mitochondrial dysfunction observed in patients with AD (27). Adiponectin modulates the expression of inflammatory molecules and protects neurons against β-amyloid toxicity (53), supporting the evidence of a link between the immune response and insulin resistance. Taken together, these findings support the role of adipokines and hormones related to the adipoinsular axis in the development of Alzheimer’s disease. Leptin and amylin can modulate the activity of key molecules involved in energy balance and neurogenesis. Ghrelin is also able to regulate not only energy metabolism but also cognitive function and active memory capacity. Adipokines are also capable to modulate the inflammatory response mediated by the glial cells. Thus, adipokines can play a relevant role as targets in the treatment of AD neurodegeneration processes and support to the relationship between AD and diabetes or MetS (26).

### AD and cardiovascular disease

Multiple lines of evidence demonstrate a strong functional linkage between cardiovascular functionality and AD-related pathophysiology (54). Amyloid peptides have been demonstrated to be directly toxic to endothelial cells in the peripheral and cerebral circulation (55, 56). Due to AD-related vascular dysfunction, local hypoxic events in the central nervous system (CNS) may further elevate metabolic dysfunction and eventual amyloid plaque deposition and NFT generation. Initial vascular damage in the CNS can also cause a significant reduction in blood–brain-barrier (BBB) integrity, which can result in pathophysiological exchange of humoral agents, toxic proteins (e.g., amyloid), or abnormal hormone levels between the periphery and CNS (57). In addition to the detrimental effects of amyloid upon vascular structures, there is also considerable evidence of AD-related cellular toxicity induced by amyloid aggregation-mediated disruption of normal protein stability, folding, and proteolysis in cardiac tissues (58).

### AD and circadian rhythms

Significant alterations in daily behavioral and sleep patterns are commonly described in AD and other neurodegenerative disorders (59). Notable disruptions of circadian rhythm in AD include the fragmentation of the sleep–wake cycle leading to increased nocturnal awakenings with increased daytime sleep bouts (60). Prospective human studies have indicated that circadian activity pattern disruption (decreased rhythm amplitude and phase-delays) are significant predictors of subsequent AD, suggesting that compromised rhythms might actually be a reliable preclinical phenomenon (61). Circadian rhythms are generated in the suprachiasmatic nucleus (SCN) as an output of the clock gene cycle, produced by a series of interlocking transcriptional feedback/feedforward loops of a panel of clock genes (e.g., PER1,2, CRY1,2, CLOCK, BMAL1) (62). Outside of the SCN, there are circadian oscillators throughout the body that control multiple autonomic functions. As the activity of many tissues is strongly regulated in a circadian manner, it is not surprising that disruption can negatively impact immune, metabolic, and cardiovascular systems (59).

### AD and sensory modalities

Neurodegeneration associated with AD affects multiple central and peripheral systems, including several perceptive modalities including vision, taste, pain, and olfaction (63–65). Olfactory impairment has been shown to be predictive of conversion from mild cognitive impairment to AD with a higher than 80% sensitivity (66). With respect to it has also been demonstrated that both cognitively impaired and AD patients present a significant reduction of total taste scores and also individual taste scores on either side of the tongue (67). As sensory modalities are currently further investigated in AD, it appears that these systems may eventually serve as potential “therapeutic gateways” into the psychosocial and physiological alterations in AD (66).

## Parkinson’s Disease

Parkinson’s disease (PD) is the most common movement disorder with an age-dependent prevalence, affecting about 1% of individuals over 60 years of age and increasing up to 4–5% at an age of 85 years (68). Studies have demonstrated a broad genetic etiology for several forms of PD and related Parkinsonian disorders (69). Pathomechanisms associated with mutations in PD-related genes involve mitochondrial dysfunction, abnormal protein degradation, and oxidative stress (70, 71). In about 95% of PD patients, however, no apparent genetic linkage (sporadic PD) has been described, indicating that PD is a multifactorial disease that arises owing to both genetic and environmental factors (69). The classical diagnosis of PD is based on the presence of bradykinesia (slowness of movement) together with at least one other cardinal motor feature such as resting tremor, rigidity, and postural instability (72, 73). Many PD patients experience also a wide range of non-motor (NM) deficiencies, including neuropsychiatric symptoms, dysautonomia (i.e., malfunction of the autonomic nervous system; ANS), hyposmia, sensory loss, sleep disturbances, and GI dysfunction. Many of these NM symptoms precede the onset of core motor dysfunctions and have a multisystem origin. Cognitive and neuropsychiatric symptoms of PD range from depression, anxiety, and apathy to frank dementia (68, 74). The occurrence of mild cognitive impairment and subsequent dementia is common in PD patients, but is more prevalent in later stages of the disease where it affects up to 80% of patients (74, 75). Two dementia types are associated with PD: Parkinson’s disease dementia (PDD) and dementia with Lewy bodies (DLB), which can be distinguished based on the onset of extrapyramidal (EP) signs. Depression is another important feature of PD and is reported in up to 45% of PD patients. Motor dysfunction in PD is closely linked to a striatonigral dopaminergic denervation and a progressive loss of dopaminergic neurons located in the substantia nigra pars compacta (SN_PC_) (76, 77). Lewy body (LB) pathology, characterized as cytoplasmic inclusions of α-synuclein in neuronal perikarya, and neurodegeneration in PD patients extends well beyond the dopaminergic striatonigral system. Additional extranigral pathology in the CNS involves noradrenergic, serotonergic, and cholinergic systems, as well as the cerebral cortex and olfactory bulb (78). Involvement of the ANS occurs at early stages of PD involving both the sympathetic and parasympathetic ganglia, and the ENS. While nigrostriatal degeneration is responsible for motor features of PD, the extranigral degeneration is thought to account for global NM symptoms of the disease (79–81). These observations have changed our traditional view of PD as being a predominant single-system disorder with selective involvement of nigrostriatal dopaminergic neurons toward a much broader multisystem LB disorder, also defined as Parkinson complex or Lewy-complex.

### PD and sensory dysfunction

A number of NM features reported in PD can be attributed to cholinergic dysfunction, e.g., altered rapid eye movement (REM) sleep, mood, cognition, and olfaction. Olfactory impairment is highly prevalent in PD and reported in approximately 95% of early-stage PD patients. Loss of smell correlates well with neurodegeneration and concomitant α-synuclein pathology in the olfactory bulb and other secondary olfactory regions (82, 83). Hyposmia occurs early in the disease progress and may precede overt motor symptoms suggesting that PD might be a primary disorder of olfaction (84). Whereas cholinergic dysfunction is suggested as a molecular basis for hyposmia, decrements in other neurotransmitter systems involving noradrenaline and serotonin production are also reported (85–87).

### PD and gastrointestinal dysfunction

Gastrointestinal dysfunction is seen as one of the earliest manifestations of PD preceding motor involvement. The GI symptoms in PD include weight loss, dysphagia, and concomitant excessive drooling, reduced salivation, nausea, constipation, and defecatory dysfunction, which reflect a deregulation of the GI motility along the entire length of the GI tract (88). Moreover, a number of the GI manifestations such as constipation are suggested to be proportional to the risk of developing the disease (89). Neuropathological studies have indicated early enteric pathology in PD in terms of LB and Lewy neurite (LN) accumulation in the dorsal motor nucleus of the vagus (DMV) and the ENS (80, 90). Importantly, the ENS shares a number of characteristics with the CNS, but is easier accessible and analyzable compared to the CNS through colonic biopsies. Therefore, the ENS offers reasonable potential for identifying novel disease markers in living patients with PD (80). In addition to the bowel dysfunctions (constipation) reported in PD, impairments in other pelvic organs including the urinary bladder and reproductive organs (also called the genitourinary system) are common NM disorders in PD (91).

### PD and sleep pathologies

Since dopamine is known to have a role in the sleep–wake cycle (92), it is not surprising that PD patients present with sleep pathologies, e.g., sleep-maintenance insomnia, and REM sleep behavior disorder (RBD) (74). The occurrence of RBD is common in PD and manifests before the onset of clinical PD (93). In these patients, decreased cholinergic innervation was reported in sleep nuclei located in the lower brainstem. Furthermore, patients with RBD are more likely to have hyposmia, cardiac sympathetic denervation, and an increased risk for developing dementia in PD (94, 95).

### Cardiovascular system dysfunction and PD

Parkinson’s disease also involves considerable cardiovascular manifestations of dysautonomias (96). More specifically, sympathetic noradrenergic denervation of the heart is an integral part of the disease and is associated with LBs in sympathetic ganglia including those that innervate the heart. The sympathetic nerve fibers in the heart are decreased in PD. Reduced cardiac uptake and concomitant sympathetic denervation is already reported in the early disease process and increases with disease duration and severity, however, the heart also receives parasympathetic input from the dorsal vagal nucleus; there is less known about parasympathetic denervation in the heart of PD patients (97–101). Another determinant that underlies the cardiovascular dysautonomia is arterial baroreflex failure. The baroreflex is the most important vasoconstrictor mechanism to buffer acute fluctuations of arterial blood pressure (ABP) that occur during changes in posture, exercise, emotion, and other conditions (102). Together, these cardiovascular deficits result in orthostatic hypotension (OH), which is present in about 30–40% of PD patients (103).

### Metabolic dysfunction and PD

Recent epidemiological and molecular genetic studies have highlighted strong links between PD and T2DM. Patients with T2DM have an increased risk of developing AD (65%) and PD (35%) (104, 105). Overlap between PD and T2DM is further strengthened by the fact that more than 60% of PD patients have impaired insulin signaling and are glucose intolerant (106, 107). This is in line with neuropathological studies of PD patients, which have shown that insulin receptors are also densely packed on dopaminergic neurons of the SN_PC_ (108). Furthermore, a number of animal and *in vitro* studies have indicated a role for insulin and glucose metabolism in the regulation of brain dopaminergic activity and firing (106). Chronic hyperglycemia is known to induce oxidative stress and concomitant production of ROS, factors implicated in T2DM and PD etiology. Moreover, energy starvation and metabolic impairment can induce the aggregation of α-synuclein in dopaminergic cells (109). Despite the marked clinical differences between PD and T2DM, an intriguing common pathogenesis is emerging and involves alterations in mitochondrial turnover, neuroinflammation, protein degradation, and glucose metabolism [for review, see Ref. (107)].

### PD and inflammation

Chronic inflammation is a prominent feature of multiple neurodegenerative disorders including PD (110, 111). Neuroinflammation is strongly associated with dopaminergic neuron degeneration and progression of PD. Leucine-rich repeat kinase 2 (LRRK2), a kinase mutated in both autosomal-dominantly inherited and sporadic PD cases, modulates inflammation in response to different pathological stimuli. PD-associated LRRK2 mutations may sensitize microglia cells toward a pro-inflammatory state, which in turn results in exacerbated inflammation with consequent neurodegeneration (112). Inflammatory pathways in PD appear to play a crucial role in the destruction of both pancreatic islet β-cells and dopaminergic neurons in the substantia nigra (113). Emerging evidence indicates that system-wide metabolic dysfunction in PD can induce metabolic inflammation, thus exacerbating the neurodegenerative activity in this disorder. The combined interaction between energy balance and inflammatory responses in PD therefore represent an important field for therapeutic study.

## Huntington’s Disease

Huntington’s disease (HD) is a disabling neurodegenerative disorder characterized by a progressive impairment of motor and cognitive functions and is caused by a mutation that takes the form of a CAG trinucleotide repeat expansion in exon 1 of the huntingtin (htt) gene on chromosome 4 (114, 115). Presently, the precise nature of the molecular functionality of endogenous non-mutant huntingtin is not comprehensively appreciated. However, huntingtin appears to be associated with modulation of BDNF expression (116), cytoskeletal organization (117), vesicle trafficking (118), and clathrin-mediated endocytic pathways (119). The mutated form, with the polyglutamine expansion, possesses an altered protein structure leading to its aggregation in the CNS. These changes in protein function and aggregation then invariably lead to neuronal degeneration. HD manifests in a variety of symptoms, which can be behavioral, motoric, and cognitive. Behavioral changes commonly occur before motor symptoms and include mood changes, irritability, restlessness, psychosis, and hallucinations. Motor symptoms mainly occur as quick sudden movements in the arms, legs, and face (chorea). Tremors, unsteady gait and head turning, and shift eye position also occur as motor symptoms. As the disease prognosis worsens, a progressive dementia occurs in the form of memory loss, disorientation, confusion, and loss of judgment. Huntington’s disease is also associated with several other debilitating non-neuronal impairments that also occur in other neurodegenerative disorders, particularly PD. These include weight loss as one of the major symptoms, insulin resistance, changes in energy metabolism, and sleep disturbance due to the disturbance of the circadian rhythm (120–122).

### HD and metabolic dysfunction

In the pre-symptomatic phase and over the course of HD progression, several disruptive changes occur to the neuroendocrine system as a result of the htt mutation. Such changes can lead to detrimental effects to appetite, body weight, mood changes, and alterations of metabolism. One of the hubs of neuroendocrine metabolic disruption in neurodegenerative disease is the hypothalamus. Proteomic analysis of rat hypothalami expressing mutant htt has demonstrated alterations of heat shock protein-70 (Hsp-70), glutathione peroxidase (Gpx4) responsible for oxidative damage, glial fibrillary acidic protein (Gfap), and the enzyme acylglycerol-3-phosphate *O*-acyltransferase 1 (Agpat1) responsible for lipid synthesis (122). In addition to these hypothalamic findings, alterations in circulating endocrine factors are apparent in HD models, i.e., reduced insulin and leptin as well as reduced triglycerides and HDL. These changes were present presymptomatically and are concordant with other human studies. Aziz et al. (123) demonstrated that there are no significant differences in the characteristics of secretion of growth hormone (GH) and ghrelin of HD patients compared to controls. With disease progression, there are significant increases in secretion and irregularity of GH combined with an increased suppression of post-prandial ghrelin suppression. Such findings indicate that the impairment of regulation of GH and ghrelin secretion is positively correlated with worsening HD prognosis. HD patients as well as murine HD models demonstrate considerable diabetic-like pathologies, which create a severe metabolic stress and energetic dysregulation (121, 124–126). HD patients are more prone to T2DM than the general population (127). Such pathologies manifest in the form of hyperglycemia and abnormal lipid metabolism. HD is linked directly with a form of dysglycemia leading to a catabolic state characterized by weight loss and a lower body mass index than the healthy population. The lipid dysregulation involves high levels of triglycerides and LDL, which also creates a risk of microvascular and macrovascular pathologies on the long (124, 125). Insulin resistance is therefore thought to be one of the main factors of the pathogenesis of HD leading to severe neurobiological impairments. This hypothesis is supported by the InCHIANTI study where people with cognitive impairments were found to be more likely to suffer insulin resistance compared to controls (128). A clear relationship has been established between insulin secretion and HD using the R6/2 mouse model. These mice have shown glycosuria and glucose intolerance while above 70% of them develop diabetes at 14 weeks (126). The bacterial artificial chromosome-mediated transgenic HD (BACHD) mouse model has also shown impaired glucose metabolism and increased resistance to insulin and leptin (diabetic-like pathology). These effects were replicated by expressing a short fragment of mutant htt using an adenoviral vector, which causes hypothalamic inactivation (129).

### HD and gastrointestinal functionality

Huntington’s disease patients have severe autonomic dysfunctions manifesting as disruptions of the GI tract, urinary and cardiovascular systems, as well as sexual dysfunction in men (114). Symptoms include difficulties of swallowing, dysphagia, early abdominal filling, defecation difficulties, urinary incontinence, and incomplete bladder emptying and postural hypotension. Such symptoms are very common in HD patients before the appearance of overt motor symptoms and also in carriers of the mutation but with less severity. Mutant htt is expressed along the GI tract and throughout the ENS. HD mouse models demonstrate a significant loss of functional neuropeptides in enteric nerves, decreased thickening of the GI mucosa, and villi length (130). Functionality of the GI tract is also significantly impaired in terms of gut motility and absorption of food. The degree of malabsorption inversely correlated with body weight indicating the importance of the GI tract dysfunction in weight loss and thus patient quality of life.

### HD and circadian rhythm alterations

Circadian studies have shown that HD patients suffer from abnormal night–day ratios and in addition the R6/2 HD mouse model also showed an abnormal night–day activity that was further disturbed as the disease progressed (131, 132). These disruptions were accompanied by an abnormal expression of clock genes in the SCN, striatum, and the motor cortex, correlating with the cognitive impairment and decline (132). In HD, it appears that functionality of the isolated SCN is specifically intact but that the pathophysiology is due to a dysfunction of the systemic circuitry rather than the SCN itself (133–135). Circadian changes in melatonin levels have been reported in HD patients (123). Synthesis of melatonin is regulated by the SCN and has a major role in the regulation of sleep as well as in peripheral circadian rhythms. Due to the ubiquitous expression of mutant htt and the presence of both central and peripheral circadian rhythms, HD-related pathology results in disruption of sleep as well as in uncoupling of peripheral and central circadian rhythms. For example, if liver metabolism is uncoupled from circadian rhythms, this leads to metabolic disruption and a detrimental effect on the disease progression (136). Levels of wakefulness-promoting factors such as orexin, ghrelin, adrenocorticotrophin hormone, and corticotrophin-releasing hormone have all been found to be abnormal in HD (136). Consequences of sleep deprivation including stress, depression, reduced immunity, memory and learning impairments, and metabolic and hormonal abnormalities are all likely to further exacerbate HD-related pathophysiology across the whole body (137–140).

### HD and cardiovascular dysfunction

Consistent with a significant autonomic dysfunction, the cardiovascular system is also impaired in HD patients as well as in murine HD models (141, 142). It appears that both sympathetic and parasympathetic cardiovascular system-regulating systems as well as the baroreceptor reflexes are impaired along with their central regulatory pathways (143). HD patients are more than 10 times more likely to suffer from cardiovascular health issues compared to normal patients (144). While a significant component of cardiovascular pathology in HD may be due to autonomic nervous disruption, recent evidence has also demonstrated a direct pathological action of mutant htt upon cardiomyocytes as well (144, 145).

### HD and inflammatory processes

Studies have provided evidence that the immune system is pathophysiologically active in HD even before overt disease manifestation (146). In addition, deficits of immune cell migration in response to chemoattractants have been obtained from human studies (147). Chemokine profiles are also significantly altered in HD patients as the disease progresses, with elevations of eotaxin and eotaxin-3, the chemokine (C–C motif) ligand 2 (CCL2), 3 (CCL3), and 4 (CCL4) (148). In addition to altered chemokine profiles, there is considerable evidence that demonstrates increased levels of circulating and CNS-borne pro-inflammatory cytokines, including IL-6, IL-8, and TNF-α (147). Reinforcing the potential importance of TNF-α in HD pathogenesis, it has recently been shown that therapeutic inhibition of TNF-α activity can significantly attenuate central and peripheral inflammation in the R6/2 HD model mice (149).

### HD and sensory modalities

As with the other major neurodegenerative disorders discussed here, HD pathophysiology has been demonstrated to impact several sensory modalities. Hence, HD disease presentation and progression has been associated with disruption of visual perceptive systems (150), impairment of olfactory sensitivity (151), and alterations in gustatory responses to multiple stimulating tastants (152). With respect to gustation, it has been hypothesized that the specific disruption of this sensory modality may be a specific early indication of the loss of sufficient neurotrophic support in the CNS (153).

## Signaling System “Super-Axes”

**Figure 1:**
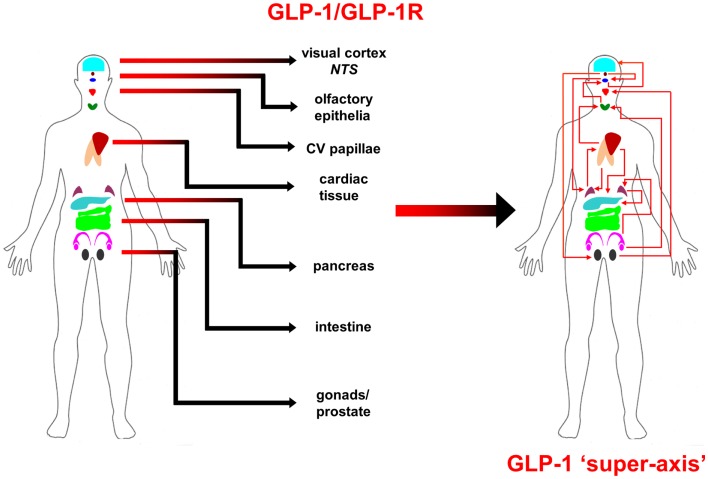
**Glucagon-like peptide 1 (GLP-1) ligand and receptor system super-axis are shown**. The expression of the ligand and receptor components of the GLP-1 system spans the whole human body. The repetitive expression of this GPCR system in multiple tissue types reinforces the importance of maintaining energy balance across the whole organism with an easily coordinated mechanism. The physical and hormonal connection between these multiple sites of GPCR functionality therefore can represent a “super-axis” of signaling connectivity that spreads across and over more classically defined tissue–tissue axes, such as the hypothalamic–pituitary– gonadotropic axis.

**Figure 2:**
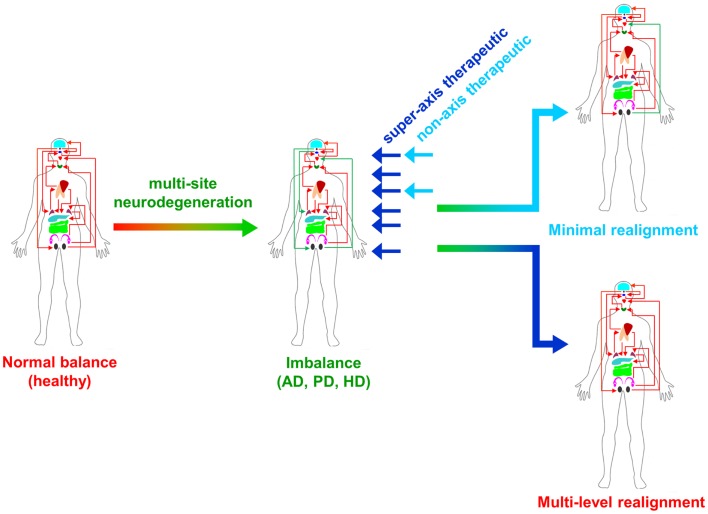
**Super-axis remediation of complex systemic disorders is shown**. Classical neurodegenerative diseases such as Alzheimer’s, Parkinson’s, and Huntington’s disease (AD, PD, and HD) represent intensely complex pathophysiological perturbations of normal systemic biology. These neurodegenerative disorders generate their full phenotypes through the disruption of multiple connected tissue–tissue signaling systems. Therapeutics that can interdict these perturbations at multiple sites in the disease process, i.e., “super-axis” therapeutics (dark blue) possess a much greater capacity to redress the systemic imbalances induced by disease than traditional non-axis therapeutics (light blue) that possess a limited functional repertoire.

From multiple sources of information, both physiological and molecular biological, it now seems apparent that for many of the major CNS neurodegenerative disorders. i.e., AD, PD, and HD, their true pathophysiological spectrum is more widespread across the body than previously considered. In addition, it is clear that there are strong similarities between these neurodegenerative conditions, suggesting perhaps that a considerable proportion of these diseases is controlled by endogenous signaling systems that are merely perturbed by the initial disease locus, but then once activated stimulate a coherent series of pathophysiological activities. This potential system-wide disease–response axis clearly needs mechanisms to maintain its activity and also coordinate its functionality across diverse tissues in varied locations across the body. As we have previously discussed in the Section “Introduction,” the evolution of receptor signaling systems has had to deal with the challenges of intense somatic development from nematode worms to the hyper-complex *Homo sapiens*. The presence of the same receptor signaling system, in multiple specialized conformations, and in diverse tissues has been demonstrated for cholinergic ligands such as acetylcholine and peptidergic ligands such as gonadotropin-releasing hormone (GnRH: 12). With respect to a receptor signaling that may be preferentially involved in regulating the generic neurodegenerative “super-axis” system, we have identified a potential ligand–receptor system, the glucagon-like peptide 1 (GLP-1) system that may be critical for regulating pathophysiology, and therefore, also facilitating potential neurodegenerative remediation. Historically, GLP-1 has been considered primarily a gut incretin that is vitally involved, in concert with insulin, with glucose metabolism. GLP-1 is produced both in pancreatic α-cells as well as intestinal L-cells (154). Upon release into the circulation after food ingestion, GLP-1 facilitates glucose uptake by directly acting on pancreatic islet β-cells to enhance post-prandial insulin secretion (155). This process is mediated by GLP-1-mediated activation of a class B1 (secretin-like family) seven transmembrane spanning GPCRs (156, 157). The GLP-1 receptor (GLP-1R) has been shown to functionally interact with both heterotrimeric G proteins [Gαs, Gαq (158) as well as β-arrestin (159)]. This promiscuity of the GLP-1R therefore facilitates the ability to flexibly stimulate this receptor system to engender multiple downstream signaling cascades (1). Underpinning our assertion that the GLP-1 signaling system may represent an organism-wide functional “super-axis,” it has been demonstrated that components of the GLP-1 system are found in multiple tissues all the way across the body from the tongue, olfactory epithelia, CNS, heart, pancreas, intestine to reproductive tissues [Figure 1 (160–166)]. Considering the vital role of GLP-1 in energy metabolism and in maintaining the viability of multiple tissues, it is unsurprising that this receptor system has been transposed across the body. This super-axis therefore creates that possibility of multi-site, multi-tissue drug remediation of neurodegenerative disorders. Given the recent emergence of appreciation of the importance of metabolic support to diseases such as AD, PD, and HD (107, 114, 122), it is evident that advanced therapeutic control of the GLP-1 super-axis could therefore generate an excellent capacity to generate whole-organism systemic therapeutic actions. With respect to the connection between neuropathophysiology, it has been demonstrated that GLP-1 signaling is critically involved in metabolic regulation (165), controlling inflammatory processes (167), regulate gut–brain axis activity (168), control multiple sensory modalities (169, 170), modulate cardiovascular activity (171), coordinate sleep–wake cycles and circadian rhythms (172, 173). The GLP-1 system is likely to be only one of the several ligand–receptor systems that exerts a super-axis level of impact upon neurodegenerative mechanisms from multiple divergent initiator loci, however, due to considerable advances in therapeutic ligand design, it does represent an important target for the creation of a systems-level remedial agent. Concordant with the findings that the GLP-1 receptor system is intimately involved in multiple aspects of the neurodegenerative axes of AD, PD, and HD, it is unsurprising that ligands that can target this receptor system have been demonstrated to exert multiple remedial and effective actions (168, 174–178). The therapeutic regulation of such systems-level receptor systems clearly represents an excellent target for more nuanced therapeutic design as the efficacy of such “super-axis” compounds may be reinforced very strongly via multiple forms of tissue-to-tissue communication. Thus, it is likely that systems-level therapies may be far more efficacious than compared to receptor-modulating ligands that are only targeted to one specific component of the neurodegenerative axis (Figure 2). In this context of the potential “super-axis” therapies, a more advanced appreciation of the functional pharmacology of these receptor–ligand systems is vital. The generation of novel tissue and/or signal-selective GLP-1 modulating agents (1, 10, 179) is therefore perhaps one of the most important future fields of study for neurodegeneration.

### GLP-1 ligand pharmacology

The GLP-1 glycoprotein hormone exists in two circulating molecular forms, GLP-1(7–37) and GLP-1(7–36) amide, both of which are full agonists at the GLP-1R (180). As stated before, the GLP-1R is expressed in multiple tissues including the central and peripheral nervous systems, heart, pancreas, kidney, lung, and GI tract. Once in the circulation, the GLP-1 peptide possesses a very short half-life (1–2 min) due to the proteolytic activity of dipeptidyl peptidase IV (DPP IV). Therefore, the creation of systemic therapeutics targeting the GLP-1R system first focused upon modification of the peptide backbone to prevent this degradation. Multiple strategies have been employed to improve the bioavailability of GLP-1 including, fatty acid acylation (181), addition of polyethylene glycol groups (182), d-amino acid substitutions (183), and transferrin hybridization (164). Perhaps, one of the most effective strategies for therapeutic generation was the creation of agents based on reptile forms of GLP-1 (exendin-4) that possess natural mutations that prevent DPP IV proteolysis (184). Exendin-4 has subsequently demonstrated multiple efficacious effects against a wide range of neurodegenerative disorders including AD, PD, and HD, reinforcing the importance of therapeutic targeting of this signaling super-axis. While the initial series of GLP-1 system-targeted therapies were peptidergically based, there is now considerable interest in the generation of novel, non-peptidergic ligands that can modify specific activities of the GLP-1 receptors (185–187). Non-peptidergic small-molecule ligands typically represent a more facile mechanism for drug production and administration. As the GLP-1R is a Class B secretin-like receptor, designing small-molecule agents that regulate the activity through an orthosteric interaction has proven difficult. However, the generation of small molecules that can allosterically interact to modulate GLP-1-mediated receptor activation has yielded interesting results, especially with regard to signal selectivity of functions. The search for small-molecule agonists/modulators of the GLP-1R has led to the identification of multiple compounds that can bind to and modulate GLP-1R function. Many of the newly developed small-molecule ligands for the GLP-1R (BETP, Novo Nordisk quinoxaline-based “Compound 2” [(6,7-dichloro2-methylsulfonyl-3-tert-butylaminoquinoxaline) (188), TT15, Boc5 (189–194)] possess intrinsic efficacy at the receptor, with respect to cAMP generation, and can augment insulin secretion either alone or in combination with peptide occupation of the GLP-1R orthosteric site. As the GLP-1R represents an interesting mechanism for controlling multiple levels of neurodegenerative behavior, it may be important for future therapeutic design to engineer the capacity for specific and beneficial tissue-based signaling bias to exploit the full therapeutic potential of this super-axis.

### GLP-1R signaling bias

For ligand–receptor systems that may control whole-organism super-axes, it is clearly important that their regulation is tightly controlled in a highly nuanced manner. Thus, the molecular regulation networks of the GLP-1 receptor system are highly complex, with multiple endogenous and exogenous peptides [at least six: GLP-1(7–36)NH_2_, GLP-1(1–36)NH_2_, GLP-1(7–37), GLP-1(1–37), GLP-1(9–36)NH_2_, and oxyntomodulin] that interact with the receptor that results in the activation of numerous downstream signaling cascades (195). In the GLP-1R super-axis system, it is clearly crucial that selectively exploiting, via signal selectivity and bias, the full signaling repertoire of the GLP-1R could have tremendous benefit for multidimensional neurodegenerative research.

Surprisingly, for such an important emerging super-axis level therapeutic target, the current molecular appreciation of GLP-1R signaling and regulation is relatively limited compared to Class I rhodopsin-like receptors. Thus, the full gamut of GLP-1-modulated signaling paradigms is still a subject of intense research. In this respect, the GLP-1R has already demonstrated a considerable degree of signaling promiscuity: coupling has been demonstrated functional interactions with multiple heterotrimeric G proteins αs, αi/o, and αq/11 (196) as well as with β-arrestin-mediated pathways (159, 197). In addition to these classical signaling mechanisms, lateral signal transfer, i.e., “transactivation” (198), from the GLP-1R to epidermal growth factor receptor has also been reported (199). With most GPCRs studied to date, there is clearly a generic capacity for the activation of multiple and diverse signaling paradigms (1). For a receptor that can interact with multiple ligands, both orthosteric and allosteric, it is highly unlikely that the identical downstream signaling behavior, when studied at a high-dimensionality level (200), can be induced by chemically distinct ligands. Therefore, among both endogenous peptidergic agents and xenobiotics, there is a tremendous capacity to identify and therapeutically engineer ligand bias at such pleiotropic receptors. An additional layer of signaling complexity is also induced by the expression of the specific GPCRs in diverse tissues that contain differing types of GPCR-interacting proteins that again add further “texture” to the eventual signal.

In recent years using differential structure–activity-relationship analysis, effective signaling bias between cAMP-related pathways (MAPK signaling and calcium mobilization) for peptidergic ligands at the GLP-1R has been demonstrated (188, 192, 201). In addition to bias at the GLP-1R of peptidergic agents, small-molecule ligands that typically interact allosterically with the GLP-1R have also demonstrated an ability to exert selective signaling actions. These allosteric receptor interactions of the small molecules can be contemporaneous with the orthosteric ligand and can affect the conformational induction or selection of the receptor. Allosteric modulators, e.g., Novo Nordisk “Compound 2” (188, 201) can demonstrate their allosteric efficacy differentially between some of the endogenous stimulatory peptides as well as for peptidergic xenobiotics as well (188, 202). The allosteric interaction of Novo Nordisk “Compound 2” was also shown to significantly alter the qualitative nature of the transduced molecular signal as well from the orthosteric peptide ligands. Such allosteric activity at the GLP-1R can also be generated by naturally occurring medicinal agents such as quercetin and catechin, which can selectively augment specific signaling activities from specific subsets of peptidergic orthosteric agents (188, 203). Other modulators such as BETP, which still exerts qualitative signal conditioning effects on orthosteric ligand activity, have also shown an intrinsic efficacy at the GLP-1R in the absence of orthosteric engagement of the receptor (201). Not only are the activities of allosteric agents dependent on the qualitative nature of the resident agent in the orthosteric site but the resultant effects of the allosteric ligand can be further conditioned by changes in receptor transport/desensitization dynamics as well as receptor scaffolding and dimerization (196, 204).

It is clear that biased signaling is a significant pharmacological aspect of the GLP-1R and that this can include both G protein-dependent and G protein-independent pathways. This situations is then further complicated with the addition of orthosteric-selective allosteric receptor modulation. Rational and targeted exploitation of these pharmacological characteristics to engender tissue- and signaling-specific activity of the GLP-1 super-axis could yield tremendous therapeutic benefit for multiple neurodegenerative disorders. The functional and pathophysiological similarities of disorders such as AD, PD, and HD, potentially generated by a commonality of energy insufficiency in each disease (27, 205–209), presents a systemic level drug target whose exploitation may create remedial activities far beyond those induced by more monopharmacological agents. Drugs that target and remediate signaling super-axes in disease will likely be more tolerable to the patient as well, as endogenous tissue-to-tissue communication pipelines will not be significantly disrupted and thus the drug activity will be less opposed by homeostatic mechanisms. Our further enhanced understanding of how multiple pathophysiological processes in the body can be connected by functional signaling super-axes will hopefully allow the creation of a new series of more effective, tolerable, and ultimately beneficial anti-neurodegenerative therapies.

## Conflict of Interest Statement

The authors declare that the research was conducted in the absence of any commercial or financial relationships that could be construed as a potential conflict of interest.
